# Variation in accumulated dose of volumetric‐modulated arc therapy for pancreatic cancer due to different beam starting phases

**DOI:** 10.1002/acm2.12720

**Published:** 2019-09-20

**Authors:** Makoto Sasaki, Mitsuhiro Nakamura, Nobutaka Mukumoto, Yoko Goto, Yoshitomo Ishihara, Manabu Nakata, Naozo Sugimoto, Takashi Mizowaki

**Affiliations:** ^1^ Human Health Sciences Graduate School of Medicine Kyoto University 53 Kawahara‐cho, Shogoin, Sakyo‐ku Kyoto 606‐8507 Japan; ^2^ Division of Clinical Radiology Service Kyoto University Hospital 54 Kawahara‐cho, Shogoin, Sakyo‐ku Kyoto 606‐8507 Japan; ^3^ Radiation Oncology and Image‐applied Therapy Graduate School of Medicine, Kyoto University 54 Kawahara‐cho, Shogoin, Sakyo‐ku Kyoto 606-8507 Japan

**Keywords:** beam starting phase, DICOM‐RT plan file, interplay effect, pancreatic cancer, VMAT

## Abstract

**Purpose:**

To assess the effects of different beam starting phases on dosimetric variations in the clinical target volume (CTV) and organs at risk (OARs), and to identify the relationship between plan complexity and the dosimetric impact of interplay effects in volumetric‐modulated arc therapy (VMAT) plans for pancreatic cancer.

**Methods:**

Single and double full‐arc VMAT plans were generated for 11 patients. A dose of 50.4 Gy in 28 fractions was prescribed to cover 50% of the planning target volume. Patient‐specific Digital Imaging and Communications in Medicine–Radiation Therapy plan files were divided into 10 files based on the respiratory phases in four‐dimensional computed tomography (4DCT) simulations. The phase‐divided VMAT plans were calculated in consideration of the beam starting phase for each arc and were then combined in the mid‐ventilation phase of 4DCT (4D plans). The dose‐volumetric parameters were compared with the calculated dose distributions without consideration of the interplay effects (3D plans). Additionally, relationships among plan parameters such as modulation complexity scores, monitor units (MUs), and dose‐volumetric parameters were evaluated.

**Results:**

Dosimetric differences in the median values associated with different beam starting phases were within ± 1.0% and ± 0.2% for the CTV and ± 0.5% and ± 0.9% for the OARs during single and double full‐arc VMAT, respectively. Significant differences caused by variations in the beam starting phases were observed only for the dose‐volumetric parameters of the CTV during single full‐arc VMAT (*P* < 0.05), associated with moderate or strong correlations between the MUs and the dosimetric differences between the 4D and 3D plans.

**Conclusions:**

The beam starting phase affected CTV dosimetric variations of single full‐arc VMAT. The use of double full‐arc VMAT mitigated this problem. However, variation in the dose delivered to OARs was not dependent on the beam starting phase, even for single full‐arc VMAT.

## 1 INTRODUCTION

The National Cancer Institute reported an estimated 55,440 new cases and 44,330 deaths from pancreatic cancer in 2018 in the United States.^1^ Although surgical resection is the only treatment associated with long‐term survival of pancreatic cancer patients, relatively few patients (approximately 15–20%) are suitable for surgery at the time of diagnosis due to locally advanced unresectable disease.^2^ For such patients, radiotherapy with chemotherapeutic agents is among the recommended treatment options.^3^

The pancreas is surrounded by radiosensitive organs at risk (OARs), such as the stomach and duodenum. Several investigators have reported that severe gastrointestinal toxicity is related to high‐dose volumes in the stomach, bowels, and duodenum.^4^, ^5^, ^6^ Intensity‐modulated radiation therapy (IMRT) and volumetric‐modulated arc therapy (VMAT) can achieve a steep dose gradient between the target volume and OARs, thus reducing the rate of gastrointestinal toxicity.^7^, ^8^

Respiratory organ motion can be problematic, however, when treating pancreatic cancer with IMRT and VMAT. Akimoto et al. quantified pancreatic tumor motion three‐dimensionally during the overall treatment course using real‐time orthogonal kV imaging, reporting intrafractional variation in the tumor position of up to 10.7 mm in the superior‐inferior direction.^9^ Under such conditions, it is well known that interplay effects between dynamic multileaf collimator (MLC) motion and target volume motion can cause a degradation of the dose distribution for single‐fraction treatment.^10^, ^11^, ^12^, ^13^, ^14^, ^15^, ^16^

Two methods can be used to explore the impact of interplay effects on IMRT and VMAT: experimental phantom studies^17^, ^18^, ^19^ and 4D dose‐calculation studies involving deformable image registration (DIR).^20^, ^21^ Although experimental phantom studies accurately compare doses delivered with or without motion, they typically assumed that respiratory motion is both regular and sinusoidal; however, this is not the case for real respiratory motion. Second, most studies employed rigid phantoms, but substantial tumor deformation can occur during human respiration.^22^ Use of a 3D gel dosimetry phantom would yield accurate dose‐volume data for nonrigid targets, but this is relatively expensive and labor‐intensive.^23^

Many researchers have conducted planning studies to investigate the impact of target volume motion on the accumulated dose distribution using IMRT and VMAT under free‐breathing conditions. Kavara et al. investigated the dosimetric impact of interplay effects in VMAT under free‐breathing conditions for pancreatic cancer patients during stereotactic body radiotherapy (SBRT)^24^ and showed that the planned dose distribution adequately represented the dose while considering the interplay effects. In the SBRT plan, the gantry was rotated slowly to create a balance between the upper limit of the gantry rotational speed, the maximum dose rate, and a large prescribed dose; it was proven that interplay effects can be experimentally minimized.^25^ In contrast, the gantry rotates at almost the maximum speed during conventional fractionated VMAT, which causes a large interplay effect.^13^

Some studies have described the relationship between the beam starting phase and interplay effects for lesions other than pancreatic cancer. Rao et al. showed that the dosimetric impact of the beam starting phase for lung lesions was larger in conventional fractionated single‐arc VMAT plans compared to SBRT in terms of single‐fraction delivery.^11^ Ehrbar et al. simulated the dosimetric impact of the beam starting phase using periodic motions reflecting the average breathing period of patients to create multiple‐arc VMAT plans for liver, adrenal gland, and lung lesions.^14^ Although it is preferable to assign a beam starting phase to the original respiratory motion of each patient, given the intrafractional variation in breathing period, this was not done.

Several studies have sought to determine the dosimetric effects of interplay according to plan complexity. Hubley et al. reported that plans exhibiting higher‐level MLC modulation were particularly susceptible to interplay effects when VMAT was used for liver SBRT.^26^ In a simulation study, Edvardsson et al. used the number of monitor units (MUs)/Gy ratio to assess plan complexity, and showed that higher complexity increased interplay effects.^15^ It is clinically important to assess the relationship between plan complexity and the dosimetric impact of interplay effects in pancreatic VMAT plans that require a high degree of intensity modulation to reduce doses to the OARs; however, previous studies did not focus on pancreatic VMAT.

The aim of this study was to assess dosimetric variations caused by interplay in terms of the beam starting phase, number of arcs, and plan complexity of conventional fractionated VMAT for pancreatic cancer.

## 2 MATERIALS AND METHODS

### 2.1 Patients

The data of 11 consecutive patients with locally advanced pancreatic cancer (median age, 71 yr; range: 64–80 yr) who underwent real‐time tumor tracking IMRT between June 2013 and June 2015 were retrospectively analyzed. The tumor was located in the pancreatic head in five patients and in the pancreatic body in six patients. As a marker, a gold coil (Visicoil; IBA, Louvain‐la‐Neuve, Belgium), 10 mm long and 0.5 or 0.75 mm in diameter, was inserted percutaneously or endoscopically into the tumor 1–2 weeks before treatment. Pancreatic tumor motions indicated by the gold coil marker were greater than 10 mm under free‐breathing conditions, as observed on orthogonal kV X‐ray fluoroscopic images.^9^ Institutional review board approval was given for this study.

### 2.2 4DCT acquisition

All patients were immobilized in the supine position (while raising their arms) using the Body Fix system (Elekta, Stockholm, Sweden). Ten respiratory four‐dimensional computed tomography (4DCT) datasets were acquired via a phase‐based sorting algorithm under free‐breathing conditions in the axial cine mode using a 16‐slice CT scanner (LightSpeed RT16; GE Healthcare, Little Chalfont, UK), and the breath signal was acquired using the Real‐time Position Management System (Varian Medical Systems, Palo Alto, CA, USA) with a sample frequency of 30 Hz. A respiratory phase of 0% corresponded to the end‐inhalation phase, and 50% to the mid‐ventilation phase between consecutive end‐inhalation phases.

### 2.3 Target volume delineation

The gross tumor volumes (GTVs) of the primary tumor and OARs, including the stomach, duodenum, intestine, liver, kidneys, and spinal cord, were manually delineated in each respiratory phase by an experienced radiation oncologist. The clinical target volume (CTV) was defined as the GTV plus a 5‐mm isotropic margin. The retro‐pancreatic space and para‐aortic lymph nodes between the root of the celiac trunk and superior mesenteric artery were also included in the CTV.

The internal target volume (ITV), combining all CTVs, was determined on the mid‐ventilation phase of 4DCT. The mid‐ventilation phase was defined as that in which the CTV was located closest to the time‐weighted mean tumor position, obtained by averaging the CTV positions derived from all 10 phases of the 4DCT. The planning target volume (PTV) was then defined by adding a 5‐mm isotropic margin to the ITV.

The mean ± *SD* of the CTV and PTV was 81.9 ± 18.2 cm^3^ (range: 53.1–111.1 cm^3^) and 188.3 ± 37.8 cm^3^ (range: 142.8–256.3 cm^3^), respectively. The overlap ratio between the PTV and stomach or duodenum was 4.9 ± 3.8% (range: 1.2–15.6%).

### 2.4 3D plans

Single and double full‐arc VMAT plans were generated for each patient using the TrueBeam STx system (Varian) operating the Eclipse software (ver. 13.7.29; Varian). The collimator angle was set to 30° for the single‐arc plans and ± 30° for the double arc plans. The TrueBeam STx was equipped with a high‐definition 120 MLC with a central leaf width of 2.5 mm. The nominal energy and maximum dose rate were 10‐MV flattened photon beams and 600 MU/min, respectively. Dose calculation was performed using the Acuros XB system (ver. 13.7.14; Varian), with a grid size of 2.5 mm for the mid‐ventilation phase of 4DCT. A dose of 50.4 Gy in 28 fractions was prescribed to cover 50% of the PTV. Dose‐volume constraints for each organ are shown in Table 1. The calculated dose distributions were labeled “3D plans”.

**Table 1:** Dose‐volume constraints for each organ in volumetric modulated arc therapy

Organ	Dose‐volume constraints
PTV	D_50%_ = 50.4 Gy (100%)
	D_95%_> 90%
	D_2%_ < 105%
Liver	V_30 Gy_ < 30%
	Mean dose < 28 Gy
Kidney	V_15 Gy_ < 45%
Stomach	D_2 cc_ < 50.4 Gy
Duodenum	D_2 cc_ < 50.4 Gy
Small Bowel	V_45 Gy_ < 45%
	D_2 cc_ < 50.4 Gy
Large Bowel	V_45 Gy_ < 50%
	D_2 cc_ < 50.4 Gy
Spinal cord + 5 mm	D_2 cc_ < 45 Gy

Abbreviations: PTV, planning target volume; D_xx%_, dose covering xx% of the volume of the organ; D_yy cc_, dose covering yy cc of the volume of the organ; V_zz Gy_, volume receiving zz Gy.

After dose calculation, one Digital Imaging and Communications in Medicine–Radiation Therapy (DICOM‐RT) plan file was obtained for each plan. This original DICOM‐RT plan file contained 178 control points (CPs) that represented the beam delivery parameters, including gantry angles, MLC positions, dose rates, and MUs per degree of gantry rotation at approximately 2° gantry angle intervals for each arc.

### 2.5 4D plans

When treating actual pancreatic cancer patients with VMAT under free‐breathing conditions, the MUs delivered at each respiratory phase are not uniform. In this study, the patient breath signal acquired during 4DCT was used to create the phase‐divided plans in consideration of the beam starting phase.

**Figure 1:**
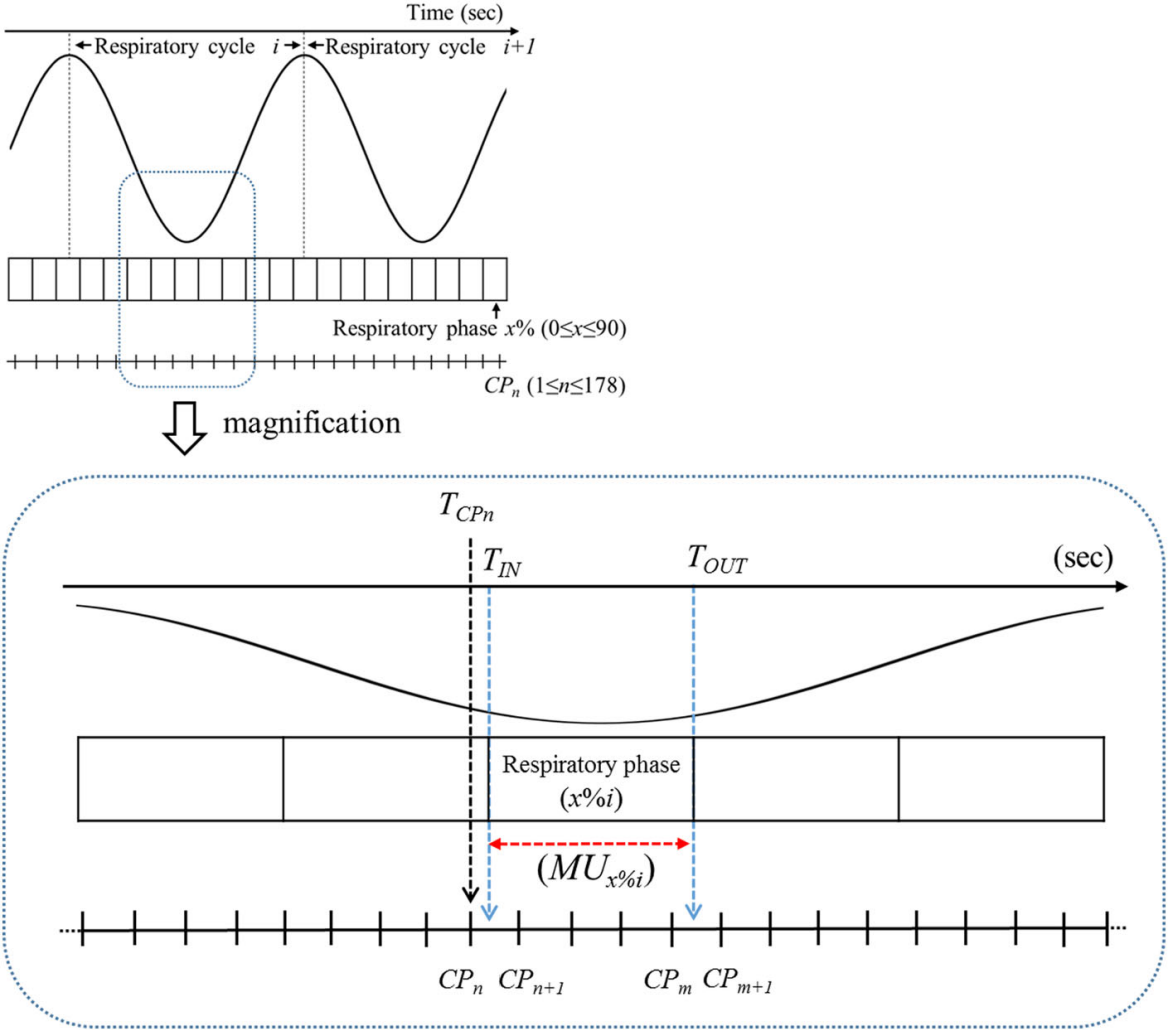
Given the MUs delivered to the *i*th part of respiratory phase *x*% (*x*%*i*), *T*_CP_ *_n_* is the time elapsed from beam delivery at a certain CP; *T*_IN_ and *T*_OUT_ are the start and end times, respectively, of the *x*%*i* extracted from the patient breath signal acquired by 4DCT, and MU*_x_*_%_ *_i_* is the MU delivered at *x*%*i*. MU, monitor unit; 4DCT, four‐dimensional computed tomography; CP, control point

Given the MUs delivered to the *i*th part of the respiratory phase *x*% (0 ≤ *x* ≤ 90) (*x*%*i*), they were determined by the interaction between the gantry rotational speeds and respiratory cycles for each arc, as follows (Fig. 1):

First, the elapsed time at a certain CP (*T*_CP_ *_n_*) from beam delivery was.

(1) $$T_{{\text{CP}}_{n}}=\left\{\begin{array}{cc} 0 & \left(n=1\right)\\ \sum_{n=2}^{178}\left(\frac{\left|{\text{GA}}_{n}-{\text{GA}}_{n-1}\right|}{{\text{GS}}_{n}}\right) & \left(2\leq n\leq 178\right) \end{array}\right.,$$

where GA and GS are the gantry angle and gantry speed recorded at a certain CP, respectively, and *n* is the ordinal number of CPs. GA recorded at the CPs was converted in the range of − 179° to 179° for clockwise gantry rotation, and in the range from 179° to − 179° for counterclockwise rotation.

Second, MU*_n_,* defined as the MU delivered between CP*_n_* and CP*_n+1_*, was given by.

(2) $${\text{MU}}_{n}=\left(\frac{\left|{\text{GA}}_{n}-{\text{GA}}_{n-1}\right|}{{\text{GS}}_{n}}\right)\times {\text{DR}}_{n}/\text{CF},$$

where DR*_n_* is the dose rate at a certain CP*_n_* and CF is a factor for converting minutes to seconds (60 s/min).

The delivery time at a certain *x*%*i*, *T*_OUT_‐*T*_IN_, was determined, where *T*_IN_ and *T*_OUT_ are the start and end times, respectively, of the *x*%*i* extracted from the patient breath signal acquired at 4DCT.

Third, MU*_x%i_* at the *x*%*i* was derived from.

(3) $${\text{MU}}_{x\%i}={\text{MU}}_{n}\times \frac{T_{{\text{CP}}_{n+1}}-T_{\text{IN}}}{T_{{\text{CP}}_{n+1}}-T_{{\text{CP}}_{n}}}+\sum_{i=n+1}^{m-1}\left({\text{MU}}_{i}\right)+{\text{MU}}_{m}\times \frac{T_{\text{OUT}}-T_{CP_{m}}}{T_{{\text{CP}}_{m+1}}-T_{{\text{CP}}_{m}}},$$

where CP*_n_* and CP*_n+1_* are the nearest CPs covering *T*_IN_; CP*_m_* and CP*_m+1_* are the nearest CPs covering *T*_OUT_; and MU*_n_* and MU*_m_* are the nearest CPs covering *T*_IN_ and *T*_OUT_, as determined by eqs. (1) and (2), respectively.

Finally, the total number of MUs delivered in the respiratory phase *x*% (*t*‐MU*_x%_*) during single full‐arc VMAT is given by.

(4) $$t-{\text{MU}}_{x\%}=\sum_{i=1}^{j}{\text{MU}}_{x\%i},$$

where *j* is the total number of parts assigned to respiratory phase *x*% during single full‐arc VMAT. These processes were repeated during the second arc of double full‐arc VMAT. In this study, the beam starting phase was given as described in the following section.

**Figure 2:**
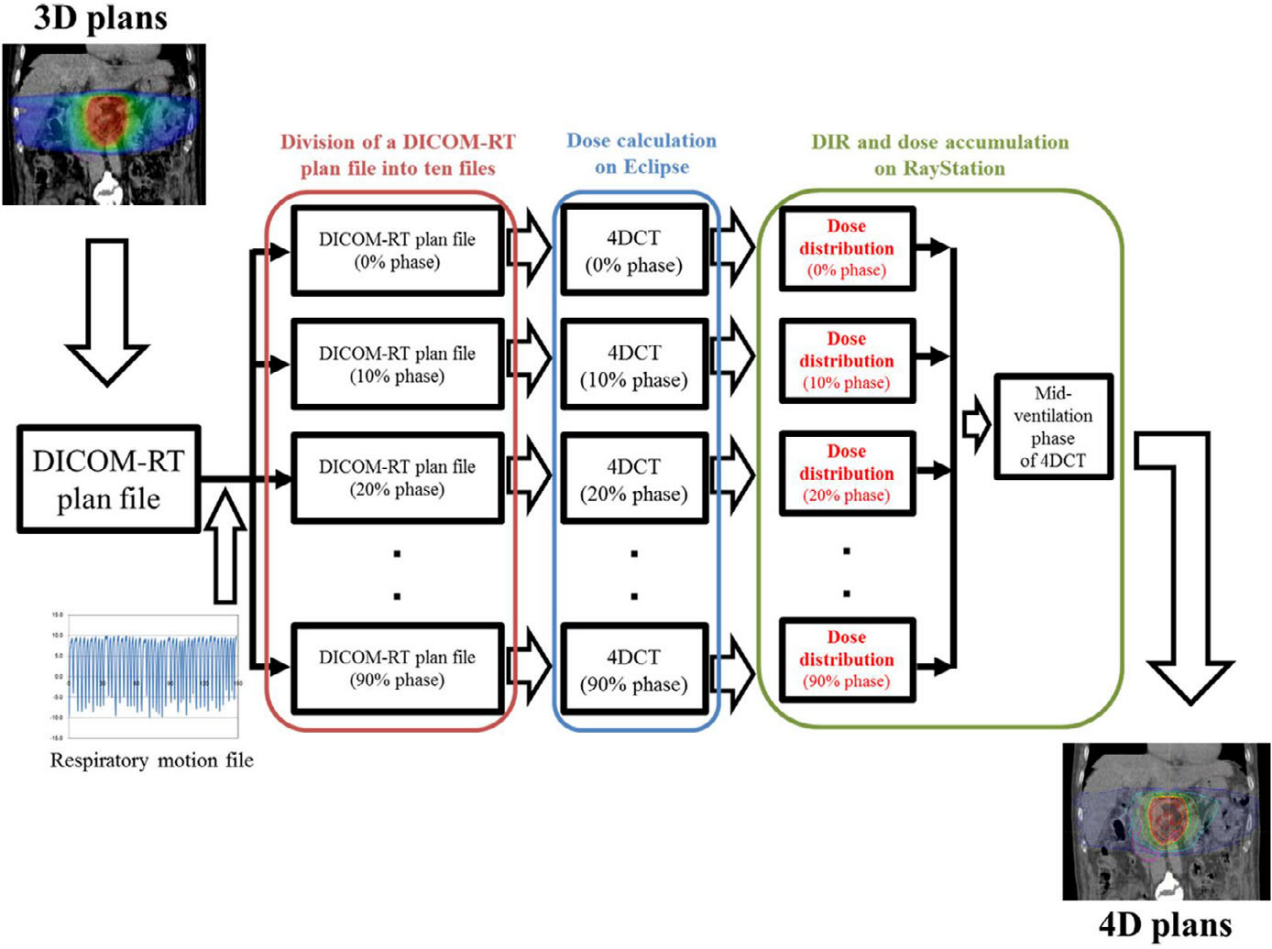
Calculation of the dose distributions in 4D plans of subdivided DICOM‐RT files, based on the respiratory phases acquired by 4DCT. DICOM‐RT, Digital Imaging and Communications in Medicine–Radiation Therapy; 4DCT, four‐dimensional computed tomography; DIR; deformable image registration

The original DICOM‐RT plan file was divided into 10 files corresponding to the respiratory phases acquired during 4DCT simulation, in consideration of the beam starting phase. Next, the 10 DICOM‐RT plan files, assigned MUs according to eq. (4), were imported into Eclipse. Subsequently, dose distributions were calculated for all 10 DICOM‐RT plan files based on the corresponding respiratory phase of 4DCT (Fig. 2) and then accumulated in the mid‐ventilation phase of 4DCT, for which 3D plans were created using the hybrid DIR algorithm ANACONDA, implemented in RayStation (ver. 6.3.0.7; RaySearch Laboratories, Stockholm, Sweden)^27^ and termed “4D plans”.

### 2.6 Evaluation

First, the gantry rotational speed and MU per degree of gantry rotation (MU/deg) were assessed in the original DICOM‐RT plan file. Modulation complexity scores (MCS) and MUs served as measures of plan complexity. MCS were then calculated from the original DICOM‐RT plan files.^28^ The MCS had values in the range 0–1, and the scores decreased when modulation increased. The total number of MUs, MU/deg, and MCS were compared using Student’s t‐test, with significance indicated by *P* < 0.05.

Second, two extreme beam starting phases of 0% and 50%, which would be associated with large dosimetric differences during respiratory cycles, were employed for single and double full‐arc VMAT. For double full‐arc VMAT, two patterns for assigning the beam starting phase to the first and second arc were then assessed; in one pattern, the same beam starting phase was assigned to two arcs (e.g., 0% or 50% for both arcs) and in the other pattern, two opposite beam starting phases were assigned (e.g., 0% for the first arc and 50% for the second arc). Prior to accumulating dose distributions, the accuracy of DIR was confirmed via visual inspection, and the uncertainty levels were scored as suggested in the AAPM Report 132.^29^ On visual inspection, registration accuracy was assigned a score of 0–4, where 0 indicated perfect registration and 4 unusable registration. The dosimetric impact of different beam starting phases was then assessed according to the dose‐volumetric parameters of the 4D plans. The Mann–Whitney U‐test and Steel–Dwass test were used to analyze the single and double full‐arc VMAT data, respectively. Significance was indicated by *P* < 0.05.

Finally, the relationship between plan complexity and dose‐volumetric parameters was evaluated using the following criteria: weak correlation, absolute correlation coefficient (|*R*|) < 0.3; moderate correlation, 0.3 ≤ |*R*| < 0.7; and strong correlation, |*R*| ≥ 0.7.

The following dose‐volumetric parameters pertaining to the prescribed dose were extracted from dose‐volume histograms (DVHs): the doses covering 98%, 50%, and 2% of the volume (D_98%_, D_50%_, and D_2%_, respectively) of the CTV, and the mean dose (D_mean_) and D_2%_ for the stomach and duodenum.

## 3 RESULTS

### 3.1 Plan information

The gantry rotational speed was constantly maintained at a maximum of 6 deg/s, except for two patients treated with single full‐arc VMAT where the gantry rotational speed at 3 (1.7%) and 8 (4.5%) CPs was less than 6 deg/s among 178 CPs. Table 2 summarizes the total number of MUs, MU/deg, and MCS. Double full‐arc VMAT plans were associated with larger MU and lower MU/deg and MCS values than were single full‐arc VMAT plans. Statistically significant differences between these metrics were observed when the single and double full‐arc VMAT plans were compared (all *P* < 0.05).

**Table 2:** Summary of MUs, MU/deg and MCS values of the 3D plan

	MUs	MU/deg	MCS
Single full‐arc VMAT	346.5 ± 29.8 (272.6–382.1)	0.97 ± 0.18 (0.51–1.88)	0.420 ± 0.045 (0.356–0.498)
Double full‐arc VMAT	407.7 ± 24.3 (334.5–424.1)	0.56 ± 0.09 (0.37–0.96)	0.354 ± 0.032 (0.282–0.423)
*P*‐value	<0.05	<0.05	<0.05

Note

The data are means ± standard deviation (range). Student’s t‐test was used to analyze the data.

Abbreviations: MU, monitor unit; MCS, modulation complexity score; VMAT, volumetric modulated arc therapy.

### 3.2 Dosimetric variations in the number of arcs, beam starting phase, and plan complexity

**Figure 3:**
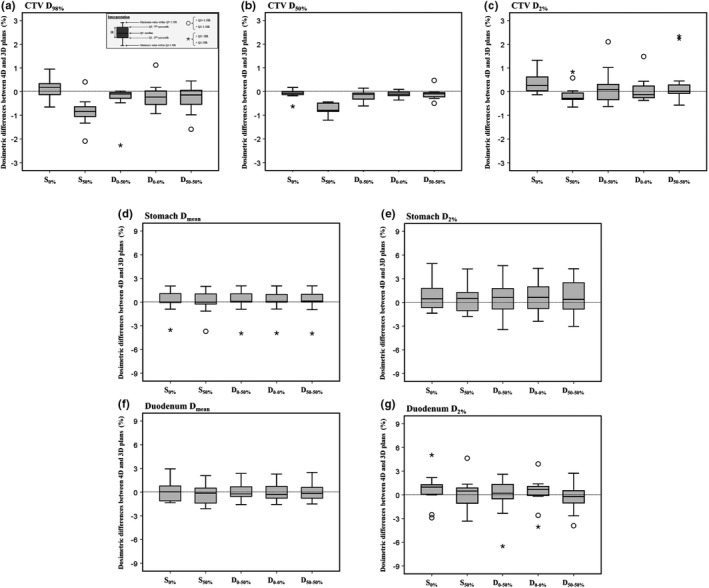
Box‐and‐whisker plots of the dosimetric differences of the CTV D_95%_ (a), D_50%_ (b), D_2%_ (c), stomach D_meam_ (d), stomach D_2%_ (e), duodenum D_mean_ (f) and duodenum D_2%_ (g) between the 4D and 3D plans by beam starting phase for single and double full‐arc VMAT plans. S_0%_, single full‐arc VMAT with a beam starting phase of 0%; S_50%_, single full‐arc VMAT with a beam starting phase of 50%; D_0–50%_, double full‐arc VMAT with beam starting phases of 0% and 50% for the first and second arcs, respectively; D_0–0%_, double full‐arc VMAT with beam starting phases of 0% for each arc; D_50–50%_, double full‐arc VMAT with beam starting phases of 50% for each arc. CTV, clinical target volume; VMAT, volumetric‐modulated arc therapy

The DIR accuracy was 1, according to AAPM Report 132.^29^ Figure 3 shows box‐and‐whisker plots of the dosimetric differences in CTV D_98%_, D_50%_, D_2%_, OAR D_mean_, and D_2%_ between the 4D and 3D plans according to the beam starting phase, for both single and double full‐arc VMAT.

The dosimetric differences for the 4D plans were consistent with those for the 3D plans, being within ± 3% of the CTV for all VMAT plans. In terms of the OARs, the differences between the 4D and 3D plans were within ± 3% in 90.9% of all plans in terms of the stomach D_mean_, and 78.2% of all plans in terms of the stomach D_2%_. The differences were within ± 3% in 100.0% of plans for the duodenum D_mean_ and 87.3% for the duodenum D_2%_.

Significant differences between the different beam starting phases were observed only in terms of the CTV dose‐volumetric parameters of single full‐arc VMAT (*P* < 0.05); however, the dosimetric differences in the median values between different beam starting phases were within ± 1.0% and ± 0.2% for the CTV and ± 0.5% and ± 0.9% for the OARs during single and double full‐arc VMAT, respectively.

Tables 3 and 4 summarize the Pearson’s correlation coefficients (*R*) for the correlations between the MCS and MUs, and dosimetric differences between the 4D and 3D plans, respectively. A moderate relationship was evident between the MCS and dosimetric variations for half of the dose‐volumetric parameters of the CTV and the OARs for single full‐arc VMAT; however, for double full‐arc VMAT there were with weak correlations with most dose‐volumetric parameters. Moderate relationships were evident between the MUs and CTV D_98%_ and D_50%_ for single full‐arc VMAT; however, for double full‐arc there were weak correlations with all dose‐volumetric parameters except for the CTV D_98%_ and D_50%_.

**Table 3:** Pearson’s correlations between the MCS and the dosimetric differences between the 4D and 3D plans with respect to the fractional doses of the dose‐volumetric parameters

Beam starting phase		CTV			Stomach		Duodenum	
		D_98%_	D_50%_	D_2%_	D_mean_	D_2%_	D_mean_	D_2%_
Single full‐arc VMAT	0%	0.09	0.57	0.22	0.47	0.51	0.13	0.45
	50%	0.31	0.48	0.23	0.46	0.51	0.20	0.21
Double full‐arc VMAT	0%–50%	0.27	0.15	0.01	0.20	0.18	0.31	0.02
	0%–0%	0.28	0.15	0.19	0.20	0.16	0.26	0.01
	50%–50%	0.37	0.27	0.05	0.19	0.21	0.22	0.02

Abbreviations: CTV, clinical target volume; MCS, modulation complex score; D_xx%_, dose covering xx% of the volume of the organ; D_mean_, mean dose.

**Table 4:** Pearson’s correlations between the MUs and the dosimetric differences between the 4D and 3D plans with respect to the fractional doses of the dose‐volumetric parameters

Beam starting phase		CTV			Stomach		Duodenum	
		D_98%_	D_50%_	D_2%_	D_mean_	D_2%_	D_mean_	D_2%_
Single full‐arc VMAT	0%	0.62	0.38	0.44	0.05	0.05	0.20	0.12
	50%	**0.79**	0.55	0.11	0.07	0.03	0.19	0.07
Double full‐arc VMAT	0%–50%	0.01	0.12	0.22	0.03	0.28	0.11	0.10
	0%–0%	0.69	0.33	0.02	0.04	0.21	0.21	0.03
	50%–50%	0.41	0.18	0.06	0.02	0.21	0.22	0.02

Note

Absolute correlation coefficients over 0.70 are shown in bold.

Abbreviations: CTV, clinical target volume; D_xx%_, dose covering xx% of the volume of the organ; D_mean_, mean dose.

## 4 DISCUSSION

In this simulation assessing the dosimetric impact of the beam starting phase in single and double full‐arc VMAT, two extreme beam starting phases, which would be expected to produce the largest dosimetric differences, were employed. In the single full‐arc VMAT plans derived using these two extreme starting phases, significant differences in the doses to the CTV were observed; however, these differences were nonsignificant during double full‐arc VMAT.

Several investigators have found that interplay effects depend on both patient‐ and machine‐specific parameters.^10^, ^15^, ^17^ We found that a dose rate reduction, achieved by increasing the number of arcs, reduced the dosimetric impact of interplay effects (Table 2 and Fig. 3), in line with the report of Court et al.^17^ In general, beam‐on time is strongly dependent on both the dose rate and beam delivery mode of non‐IMRT/VMAT. When delivering VMAT via Varian machines, however, the dose rate is controlled so that the gantry rotates at maximum speed. The rotational speed will not change according to the beam delivery mode for small fractional doses; therefore, increasing the number of arcs from one to two effectively reduces the dosimetric impact of interplay effects in conventional fractionated VMAT plans. However, extended beam‐on time may cause baseline drift,^9^ triggering interplay effects.

Edvardsson et al. found that the CTV size and collimator angle affected the interplay effects.^15^ In terms of CTV size, it was concluded that the interplay effects varied considerably by the initial breathing phase; larger variations were observed for smaller CTVs (diameters of 1 and 3 cm). Our CTVs ranged in size from 53.1 to 111.1 cm^3^, corresponding to diameters of more than 4 cm. We employed a collimator angle of 30°, as generally used in clinical practice. An earlier report found only small differences by collimator angle.^15^ Thus, any effect of dose variation will be smaller in this study than the effects of other parameters.

Several phantom and planning studies have demonstrated that interplay effects became noticeable when tumors moved largely, and when radiation was delivered over longer periods; however, delivered doses are of course averaged out over many treatment fractions.^11^, ^12^, ^13^ In this study, the dosimetric variation attributable to different beam starting phases in single‐fraction single full‐arc VMAT was up to 1.8% for the CTV. However, differences in beam starting phase and number of arcs did not result in dosimetric differences with respect to the OARs (Fig. 3), implying that the OARs were irradiated more randomly during breathing than was the CTV. The dosimetric differences between 4D and 3D plans relative to the fractional dose sometimes exceeded 3%; however, most dosimetric differences were no more than ± 8 cGy within a single fraction. Such errors would be clinically negligible over a total of 28 fractions. However, hypofractionated radiotherapy is becoming increasingly popular; the average effects are not as pronounced.^15^ There is a risk that a biased starting phase will be selected during hypofractionated radiotherapy without motion management; therefore, the selection of a consistent starting phase from the time of CT simulation to the final day of treatment, or appropriate use of respiratory motion management, such as breath‐holding or respiratory gating, is required.

DIR accuracy is never perfect due to inherent non‐negligible uncertainties.^30^ Ziegler et al. reported difficulty in evaluating registration quality because standard metrics for image comparisons, such as root mean square and feature extraction, cannot be used^31^; therefore, we assessed DIR accuracy visually. Visual inspection yielded a registration uncertainty of 1, indicating that localization was appropriate provided the target was in the locally aligned region.^29^ Calculation of the cumulative dose based on the hybrid DIR of RayStation may be very accurate even when there is organ movement.^32^, ^33^ Thus, we considered dose accumulation calculated by the DIR to be reliable.

The degree of intensity modulation was generally low in lung SBRT with VMAT, which minimized the dosimetric impact due to interplay effects.^11^ McNiven et al. reported that plan complexity increased with the complexity of the target area surrounding OARs.^34^ In general, the pancreas is surrounded by several radiosensitive OARs. In this study, up to 15.6% of the PTV overlapped with OARs. Thus, we assumed that the dosimetric impact of interplay effects could be estimated from the metrics for plan complexity, such as the MCS and MUs. The degree of intensity modulation for pancreatic cancer in the conventional fractionated VMAT plan is generally large in various disease sites.^28^, ^34^ As shown in Table 3, moderate correlations (0.3 ≤ |*R*| < 0.7) between the MCS and the dosimetric variations were more commonly observed for single rather than double full‐arc plans. In addition, moderate or strong correlations were found between MUs and dosimetric differences with respect to the CTV of single full‐arc VMAT plans (Table 4), supporting Court et al.^16^ Thus, the MCS and MUs may predict dose variations attributable to different beam starting phases.

Several limitations of this study warrant discussion. First, only two extreme beam starting phases, 0 and 50%, were used. Prior to the study, we used mid‐respiratory phases (e.g., 20 and 80%) as beam starting phases for patients exhibiting the largest dose differences to the CTV, stomach, and duodenum; we used beam starting phases of 0 or 50% for the 4D and 3D single‐arc VMAT plans. However, we found that the dose differences using beam starting phases of 20 and 80% did not exceed those associated with beam starting phases of 0 and 50%; therefore, we decided to employ the two extreme beam starting phases of 0 and 50% in this study. Second, only one 4DCT respiratory phase acquired during treatment planning was used. If the respiratory motion of a CT simulation was repeated perfectly during treatment, doses would be delivered as planned because the dose calculation algorithm is efficient, and the Linac dosimetric accuracy and calibration are sufficient.^35^, ^36^ As reported by Akimoto et al.,^9^ we found variations in motion amplitude and baseline drift during beam delivery. Also, geometric changes in internal organs can develop over the course of treatment.^6^, ^37^ Thus, intra‐ and inter‐fractional dose variations are caused by internal organ motion.

## 5 CONCLUSION

We assessed dosimetric variation in the CTV and OARs in single and double full‐arc VMAT plans under free‐breathing conditions in pancreatic cancer patients by assigning a beam starting phase to each arc. The degree of plan complexity of double full‐arc VMAT was not reduced compared to that of single full‐arc VMAT. The beam starting phase was associated with CTV dosimetric variations during single full‐arc VMAT. The use of double full‐arc VMAT reduced the influence of this factor. Meanwhile, the variation in dose delivered to OARs was not dependent on the beam starting phase, even during single full‐arc VMAT. In addition, moderate to strong correlations were observed between the MUs and dosimetric differences between 4D and 3D plans, in terms of the CTV dose‐volumetric parameters of single full‐arc VMAT.

## CONFLICT OF INTEREST

No author has any conflict of interest.

## FUNDING

This work was supported in part by JSPS KAKENHI (Grant nos. 18H02766 and 18K15630).
